# Is it possible to optimize the protein production yield by the generation of homomultimeric fusion enzymes?

**DOI:** 10.1186/s40064-016-1968-0

**Published:** 2016-03-11

**Authors:** Iryna Barshakh, Skander Elleuche

**Affiliations:** Institute for Technical Microbiology, Hamburg University of Technology (TUHH), Kasernenstr. 12, 21073 Hamburg, Germany

**Keywords:** Gene fusion, Endoglucanase, LE-cloning, Protein yield, Thermozymes, Stability

## Abstract

**Background:**

The supply of industrially relevant biocatalysts demands an easy and efficient protein production in high yield. In a conventional approach, a recombinant protein is produced in a heterologous host enabling the manipulation of multiple parameters including expression plasmids, growth conditions and regulation of protein biosynthesis. In this study, the generation of homomultimeric fusion genes is tested as an additional parameter to increase the production yield of a heat-stable cellulase.

**Findings:**

The LE (*Lgu*I/*Eco*81I)-cloning strategy was used to generate a set of plasmids containing a single copy or two to four repetitions of the endoglucanase-encoding gene *cel5A* from the thermophilic anaerobe *Fervidobacterium gondwanense*. Serial up-scaling of shaking flask volumes from 50 to 500 mL were used to determine the production yield of active cellulolytic enzyme Cel5A in recombinant form in *Escherichia coli*. Monitoring the cellular wet weight and total protein proved that the bacterial growth rate is not depending on the production of fusion enzymes, however activity assays in combination with Western blotting analyses indicated instability effects of large homomultimeric fusion enzymes.

**Conclusion:**

The production yield of fusion cellulases is constant with increasing molecular weights, but improved activities were not observed for recombinant Cel5A homomultimers. This strategy may serve as a starting point for further studies to generate more stable fusion proteins with improved catalytic activities and higher protein yield in the future.

**Electronic supplementary material:**

The online version of this article (doi:10.1186/s40064-016-1968-0) contains supplementary material, which is available to authorized users.

## Findings

The ability to express a heterologous gene and the production of its encoded protein in high yield is a prerequisite to be used in basic research and industrial processes (Rosano and Ceccarelli 2014; Tripathi et al. 2009). Extensive research has been undertaken to develop novel tools including expression plasmids, engineered strains and cultivation strategies, for the well-adapted production of individual proteins (Chen et al. 2016; Liebl et al. 2014; Makino et al. 2011; Sivashanmugam et al. 2009). Nowadays, *Escherichia coli* is probably the predominant and most popular model in terms of optimized production of recombinant proteins in academia, while filamentous fungi, yeasts and further bacteria, such as *Bacillus* spp. and *Streptomyces* spp. are dominating industrial production approaches. *E. coli* is easy to manipulate and to cultivate and allows the production of proteins for purification and characterization from foreign sources, including eukaryotes and prokaryotes from extreme environments (Elleuche et al. 2015; Sivashanmugam et al. 2009; Tripathi et al. 2009). Several strategies were pursued to increase the yield of a recombinant protein, including promoter regulation and induction of transcription, utilization of multi-copy plasmids, dual expression of two genes in a single vector and optimization of incubation conditions to name a few (Horn et al. 1996; Rosano and Ceccarelli 2014; Ma et al. 2015).

In this study, the effect of multiple identical copies of a certain gene is investigated by generating artificial homomultimeric fusion enzymes. In contrast to polycistronic operons, whose transcription and translation would result in separated proteins, fusion genes are preceded by a promoter region and a singular RBS and flanked by Start- and Stop-signals to provide a reliable context for translation of the complete ORF in a single step (Tan 2001; Rizk et al. 2012). Fusion proteins unite several advantages including the supply of multifunctional enzyme chimeras (by fusing different genes) in a single production step, instead of generating several enzyme-encoding plasmids and engineering individual strains to produce versatile proteins (Elleuche 2015; Rizk et al. 2015). As a proof-of-principle, the endoglucanase-encoding gene *cel5A* from the anaerobe thermophile *Fervidobacterium gondwanense* was chosen, because thermozymes are heat-stable, robust and enable easy handling under laboratory conditions (Elleuche et al. 2015). Moreover, this enzyme already displayed optimal characteristics and properties to be easily studied and tolerated fusions at the N- and C-terminal ends (Marquardt et al. 2014; Neddersen and Elleuche 2015; Rizk et al. 2015, 2016).

### Generation of homomultimeric fusion endoglucanases

The LE-cloning system has been developed to ligate two or more genes into a vector system, thereby enabling the reliable and easy production and purification of multifunctional biomass degrading fusion enzymes (Marquardt et al. 2014; Neddersen and Elleuche 2015). The prototype vector pQE-30-LE is based on the medium-copy plasmid pQE-30 (Qiagen, Hilden, Germany; utilization of ColE1 origin of replication results in 15–20 copies of plasmids in a single cell) that contains a T5-promoter and a sequence encoding the N-terminal HIS_6_-tag. Moreover, this vector is adapted to be optimally used in combination with expression strain *E. coli* M15[pREP4]. In addition, the MCS was replaced in pQE-30-LE by a merged recognition site for restriction endonucleases *Lgu*I and *Eco*81I to allow step-wise ligation of DNA-fragments into a continuously growing plasmid (Marquardt et al. 2014).

**Fig. 1:**
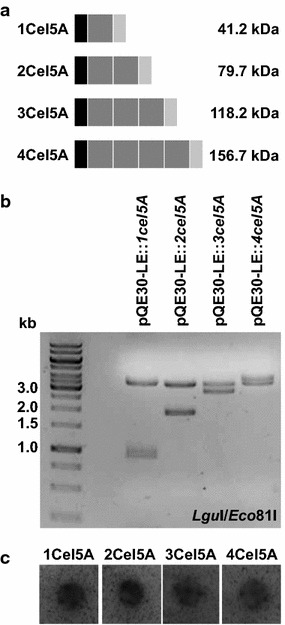
Schematic diagram illustrating fusion enzymes, cloning strategy, and enzymatic activity. **a** Schemes displaying homomultimeric fusion constructs. *Black boxes* indicate HIS-tags, *dark grey boxes* highlight Cel5A and STREP-tags are given in *light grey*. Molecular weights are indicated aside. **b** Restriction analyses of *cel5A*-encoding plasmids using *Lgu*I and *Eco*81I. **c** Qualitative plate activity assays with AZCL-HE cellulose used as substrate

Plasmids pQE-30-LE::*1cel5A* and pQE-30-LE::*2cel5A* containing a single copy of gene *cel5A* or two identical copies were obtained from proof-of-principle experiments in a previous study (Marquardt et al. 2014). ORFs *1cel5A* and *2cel5A* were excised with *Lgu*I and *Eco*81I restriction enzymes and ligated into *Eco*81I-linearized vector pQE-30-LE::*2cel5A* to give plasmids pQE-30-LE::*3cel5A* and pQE-30-LE::*4cel5A*, respectively (Additional file 1; Fig. 1a). All plasmids were tested by restriction analyses using endonucleases *Lgu*I and *Eco*81I (Fig. 1b). Furthermore, catalytic functionality was investigated by expressing the singular gene and fusion genes in *E. coli* M15[pREP4] used as a host. LB-medium plates supplemented with 50 µg/ml kanamycin, 100 µg/ml ampicillin and 0.1 mM IPTG were overlaid with AZCL-HE-cellulose containing agarose to detect enzymatic activity (Fig. 1c).

### Up-scaling the protein production in 50, 100, 250 and 500 mL shaking flasks

**Fig. 2:**
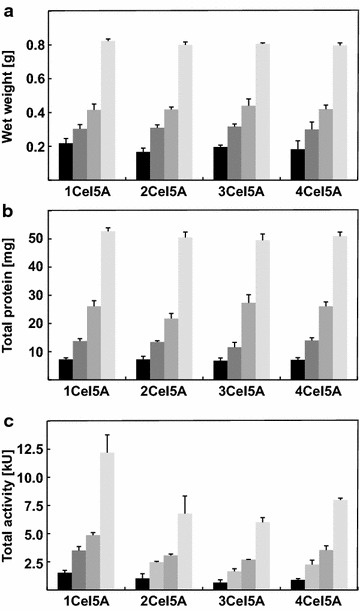
Wet weights, total protein amounts and total activities of homomultimeric fusion enzymes. **a** Heterologous *E. coli* strains were grown in different incubation volumes and produced cell masses were determined. **b** Cells were disrupted by sonication, and insoluble proteins were sedimented. Concentration of separated soluble proteins was measured. **c** Total protein activities of soluble crude protein extracts were determined with the DNS-assay. Incubation volumes are indicated by the following colour code: 50 mL *black*, 100 mL *dark grey*, 250 mL *middle grey*, 500 mL *light grey*. *Error bars* indicate standard deviations of two to six independent measurements. Raw data from all measurements are given in Additional file 2

Expression of *cel5A* from plasmid pQE-30-LE::*1cel5A* was already achieved in our previous studies and could be successfully used for further investigations including SDS-PAGE, Western blotting analyses and activity assays (Marquardt et al. 2014; Neddersen and Elleuche 2015). In the presented study, 50, 100, 250 and 500 mL shaking flasks were used for scale-up experiments. To exclusively focus on the repetition of the endoglucanase-encoding gene, all expression tests were done under identical standard conditions in LB-medium [1 % (w/v) tryptone, 0.5 % (w/v) yeast extract, 1 % (w/v) NaCl, adjusted to pH 7.2]. A defined volume of a preincubation culture (1/1000 of target culture) was used to inoculate flasks that were further incubated under constant shaking (160 rpm) at 37 °C until an optical density OD_600_ = 0.6–0.7 was reached. Gene expression was induced with 0.5 mM IPTG and cells were harvested after 4 h of incubation. All experiments were done in duplicate to sextuplicate. Monitoring the cellular wet weight revealed that a similar amount of cells (up to 0.8 g per 500 mL) expressing various endoglucanase constructs were produced at each individual incubation volume (Fig. 2a). Subsequently, cells were disrupted by sonication and concentrations of soluble proteins in the crude extracts were determined using the Bradford protein assay (Bradford 1976). In good agreements with cellular wet weights, total protein concentrations in the supernatant were similar in heterologous hosts producing different sized fusion constructs (Fig. 2b).

### Production of homomultimeric fusion enzymes leads to reduced activities

Total enzymatic activity from crude protein extracts was determined (Fig. 2c). Catalytic activities with β-glucan used as substrate were measured with the DNS-assay as described previously (Bailey 1988; Neddersen and Elleuche 2015). The activity of fusion enzymes is reduced when compared to the singular enzyme. There might be several reasons for the lowered catalytic performance including disadvantageous and improper folding in large fusion enzymes. In addition, the enlarged fusion proteins might be less soluble leading to the formation of inclusion bodies.

**Fig. 3:**
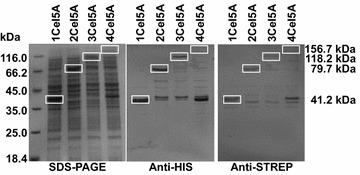
SDS-PAGE and Western blotting analyses of homomultimeric fusion enzymes. Crude protein extracts from *E. coli* M15[pREP4] LB-medium expressing *cel5A* or fusion genes were separated on SDS-PAGE and visualized by Coomassie Brilliant Blue staining. Degradation products of homomultimeric fusion enzymes were detected using Western blotting analyses with specific antibodies for terminal affinity tags. Full length proteins are *boxed in white*. Cells were grown in 500 mL and harvested 4 h after induction with 0.5 mM IPTG

To investigate these effects in more detail, further experiments were undertaken. SDS-PAGE analyses of sedimented pellet fractions in comparison with crude proteins in the supernatant revealed that all constructs were predominantly present in soluble form (Additional file 3). Total cellular proteins (insoluble and soluble) produced in *E. coli* M15[pREP4] were visualized on SDS-PAGEs and Western blots using either His-Tag^®^ Monoclonal Antibody or Strep-Tag^®^ II Monoclonal Antibody in combination with a Goat Anti-mouse FgG AP conjugate (KGaA, Darmstadt, Germany) (Fig. 3). The obtained signals are in good agreement with calculated molecular masses: Cel5A—41.2 kDa, 2Cel5A—79.7 kDa, 3Cel5A—118.2 kDa and 4Cel5A—156.7 kDa, but additional signals indicate that fusion enzymes were partly degraded. Interestingly, major degradation products displayed a comparable molecular weight (approx. 42 kDa) like the singular protein Cel5A. It is important to note that only terminal degradation products that contain an affinity tag were detectable in these Western blotting analyses, while internal parts of the proteins were not visualized. Nevertheless, breakage of the fusion enzymes at the linked regions might not come along with reduced activities, because degraded singular Cel5A moieties could restore activity. Therefore, the reduced catalytic activities in homomultimeric fusion enzymes are probably derived from folding issues, which would be in good agreement with previous observations in other studies (Hong et al. 2006, 2007; Neddersen and Elleuche 2015).

### Future directions

A major disadvantage of fusion enzymes always is the molecular weight of the final constructs that are often too large to be stably kept in the heterologous host and quickly become degraded. Although, Cel5A is a robust and globular protein, fusion leads to protein instability and reduced functional product. However, this strategy might be useful to produce increased amounts of small proteins. It has been shown before that a trimeric fusion of a cellulose-binding module (pQE-30-LE::*3cbm*) was produced in stable form with the pQE-30-LE system, but no functionality tests were done with this model protein so far (Marquardt et al. 2014). Finally, it is an important observation that the production of homomultimeric fusion enzymes did not lead to a decreased growth rate of *E. coli* in these experiments and further improvement including monitoring of transcription and translation levels may help to produce stable (and small) homomultimeric fusion proteins in high yield in the future.

## Additional files

10.1186/s40064-016-1968-0 Plasmids used in this study.
10.1186/s40064-016-1968-0 Raw data of cellular wet weights, protein production yields and activities.
10.1186/s40064-016-1968-0 Investigation of soluble and insoluble proteins.

## Abbreviations

AZCL-HE: azurine cross-linked hydroxyethyl
DNS: 3,5-dinitrosalicylic
IPTG: isopropyl-β-d-1-thiogalactopyranoside
LB: Luria Bertani
MCS: multiple cloning site
ORF: open reading frame
RBS: ribosome binding site
SDS-PAGE: sodium dodecyl sulfate polyacrylamide gel electrophoresis

## References

[CR1] Bailey MJ (1988). A note on the use of dinitrosalicylic acid for determining the products of enzymatic reactions. Appl Microbiol Biotechnol.

[CR2] Bradford MM (1976). A rapid and sensitive method for the quantitation of microgram quantities of protein utilizing the principle of protein-dye binding. Anal Biochem.

[CR3] Chen A, Sun Y, Zhang W, Peng F, Zhan C, Liu M, Yang Y, Bai Z (2016). Downsizing a pullulanase to a small molecule with improved soluble expression and secretion efficiency in *Escherichia coli*. Microb Cell Fact.

[CR4] Elleuche S (2015). Bringing functions together with fusion enzymes—from nature’s inventions to biotechnological applications. Appl Microbiol Biotechnol.

[CR5] Elleuche S, Schäfers C, Blank S, Schröder C, Antranikian G (2015). Exploration of extremophiles for high temperature biotechnological processes. Curr Opin Microbiol.

[CR6] Hong SY, Lee JS, Cho KM, Math RK, Kim YH, Hong SJ, Cho YU, Kim H, Yun HD (2006). Assembling a novel bifunctional cellulase–xylanase from *Thermotoga maritima* by end-to-end fusion. Biotechnol Lett.

[CR7] Hong SY, Lee JS, Cho KM, Math RK, Kim YH, Hong SJ, Cho YU, Cho SJ, Kim H, Yun HD (2007). Construction of the bifunctional enzyme cellulase-beta-glucosidase from the hyperthermophilic bacterium *Thermotoga maritima*. Biotechnol Lett.

[CR8] Horn U, Strittmatter W, Krebber A, Knupfer U, Kujau M, Wenderoth R, Muller K, Matzku S, Pluckthun A, Riesenberg D (1996). High volumetric yields of functional dimeric miniantibodies in *Escherichia coli*, using an optimized expression vector and high-cell-density fermentation under non-limited growth conditions. Appl Microbiol Biotechnol.

[CR9] Liebl W, Angelov A, Juergensen J, Chow J, Loeschcke A, Drepper T, Classen T, Pietruszka J, Ehrenreich A, Streit WR, Jaeger KE (2014). Alternative hosts for functional (meta)genome analysis. Appl Microbiol Biotechnol.

[CR10] Ma W, Cao W, Zhang H, Chen K, Li Y, Ouyang P (2015). Enhanced cadaverine production from l-lysine using recombinant *Escherichia coli* co-overexpressing CadA and CadB. Biotechnol Lett.

[CR11] Makino T, Skretas G, Georgiou G (2011). Strain engineering for improved expression of recombinant proteins in bacteria. Microb Cell Fact.

[CR12] Marquardt T, von der Heyde A, Elleuche S (2014). Design and establishment of a vector system that enables production of multifusion proteins and easy purification by a two-step affinity chromatography approach. J Microbiol Methods.

[CR13] Neddersen M, Elleuche S (2015). Fast and reliable production, purification and characterization of heat-stable, bifunctional enzyme chimeras. AMB Express.

[CR14] Rizk M, Antranikian G, Elleuche S (2012). End-to-end gene fusions and their impact on the production of multifunctional biomass degrading enzymes. Biochem Biophys Res Commun.

[CR15] Rizk M, Elleuche S, Antranikian G (2015). Generating bifunctional fusion enzymes composed of heat-active endoglucanase (Cel5A) and endoxylanase (XylT). Biotechnol Lett.

[CR16] Rizk M, Antranikian G, Elleuche S (2016). Influence of linker length variations on the biomass-degrading performance of heat-active enzyme chimeras. Mol Biotechnol.

[CR17] Rosano GL, Ceccarelli EA (2014). Recombinant protein expression in *Escherichia coli*: advances and challenges. Front Microbiol.

[CR18] Sivashanmugam A, Murray V, Cui C, Zhang Y, Wang J, Li Q (2009). Practical protocols for production of very high yields of recombinant proteins using *Escherichia coli*. Protein Sci.

[CR19] Tan S (2001). A modular polycistronic expression system for overexpressing protein complexes in *Escherichia coli*. Protein Expr Purif.

[CR20] Tripathi NK, Sathyaseelan K, Jana AM, Rao PVL (2009). High yield production of heterologous proteins with *Escherichia coli*. Defence Sci J.

