# Supplementary material for: Is it possible to optimize the protein production yield by the generation of homomultimeric fusion enzymes?
Source: Springerplus. 2016 Mar 11;5:316. doi: 10.1186/s40064-016-1968-0 (PMC4788654; doi:10.1186/s40064-016-1968-0)
Supplement: Supplementary file 1 — 10.1186/s40064-016-1968-0 Plasmids used in this study. [file 40064_2016_1968_MOESM1_ESM.pdf]

Additional file 1 – Plasmids used in this study

Plasmid	Characteristics	Reference
pQE-30-LE::*1cel5A*	HIS-Cel5A-STREP	(Marquardt et al. 2014)
pQE-30-LE::*2cel5A*	HIS-Cel5A-Cel5A-STREP	(Marquardt et al. 2014)
pQE-30-LE::*3cel5A*	HIS-Cel5A-Cel5A-Cel5A-STREP	This study
pQE-30-LE::*4cel5A*	HIS-Cel5A-Cel5A-Cel5A-Cel5A-STREP	This study
