# Supplementary material for: Is it possible to optimize the protein production yield by the generation of homomultimeric fusion enzymes?
Source: Springerplus. 2016 Mar 11;5:316. doi: 10.1186/s40064-016-1968-0 (PMC4788654; doi:10.1186/s40064-016-1968-0)
Supplement: Supplementary file 2 — 10.1186/s40064-016-1968-0 Raw data of cellular wet weights, protein production yields and activities. [file 40064_2016_1968_MOESM2_ESM.pdf]

Additional File 2 – Raw data of cellular wet weights, protein production yields and activities.

Cellular wet weight [g]*				
Shaking flasks	50 mL	100 mL	250 mL	500 mL
1Cel5A	0.22 ± 0.03	0.30 ± 0.03	0.42 ± 0.04	0.82 ± 0.01
2Cel5A	0.17 ± 0.02	0.31 ± 0.02	0.42 ± 0.02	0.80 ± 0.02
3Cel5A	0.20 ± 0.01	0.32 ± 0.02	0.44 ± 0.04	0.81 ± 0.01
4Cel5A	0.18 ± 0.05	0.30 ± 0.04	0.42 ± 0.02	0.80 ± 0.02
Total protein [mg]*				
Shaking flasks	50 mL	100 mL	250 mL	500 mL
1Cel5A	7.21 ± 0.56	13.71 ± 0.89	26.07 ± 1.98	52.70 ± 1.21
2Cel5A	7.22 ± 1.12	13.43 ± 0.44	21.74 ± 1.81	50.48 ± 2.00
3Cel5A	6.76 ± 0.96	11.56 ± 1.74	27.30 ± 2.91	49.49 ± 2.29
4Cel5A	7.10 ± 0.73	13.94 ± 0.96	26.07 ± 1.57	50.90 ± 1.53
Total activity [U]*				
Shaking flasks	50 mL	100 mL	250 mL	500 mL
1Cel5A	1,536.0 ± 211.0	3,507.5 ± 356.2	4,869.5 ± 233.3	12,190.7 ± 1584.6
2Cel5A	1,030.3 ± 428.1	2,470.4 ± 69.4	3,067.4 ± 128.6	6,782.6 ± 1,559.5
3Cel5A	657.9 ± 252.0	1,655,0 ± 233.1	2,695.3 ± 25.2	6,015.4 ± 406.7
4Cel5A	906.9 ± 110.4	2,261.1 ± 389.3	3,545.3 ± 377.7	7,989.7 ± 165.8

*Data were used to generate diagrams in Figure 2. Each experiment was done in duplicate to sextuplicate.
