# Supplementary material for: Is it possible to optimize the protein production yield by the generation of homomultimeric fusion enzymes?
Source: Springerplus. 2016 Mar 11;5:316. doi: 10.1186/s40064-016-1968-0 (PMC4788654; doi:10.1186/s40064-016-1968-0)
Supplement: Supplementary file 3 — 10.1186/s40064-016-1968-0 Investigation of soluble and insoluble proteins. [file 40064_2016_1968_MOESM3_ESM.pdf]

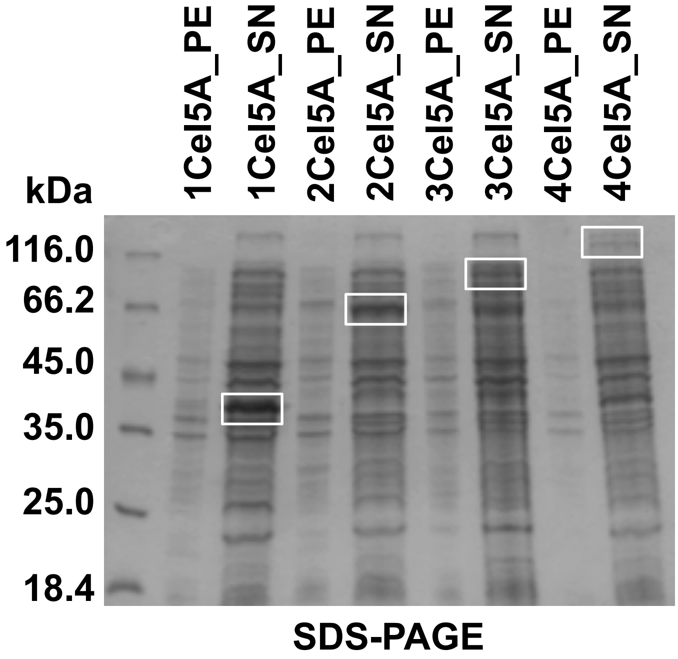

Additional File 3 – Investigation of soluble and insoluble proteins. Cel5A produced as singular or homomultimeric fusion enzymes are soluble when produced in *E. coli* M15[pREP4]. Cells were grown in 500 mL and harvested 4 h after induction with 0.5 mM IPTG. Cells were disrupted by sonication and insoluble protein aggregates were separated from soluble proteins by centrifugation. Abbreviations are as follows: PE – Pellet, insoluble fraction, SN - Supernatant
